# First Bacteremia Due to *Corynebacterium gottingense* in an Immunocompromised Child: A Case Report, 16S rDNA-Based Phylogenetic Analyses and Review of the Literature

**DOI:** 10.3390/antibiotics12030528

**Published:** 2023-03-06

**Authors:** Lucas Bouguerra, Chrystelle Dupraz, Chloé Plouzeau, Anthony Michaud, Lauranne Broutin, Julie Cremniter, Christophe Burucoa, Maxime Pichon

**Affiliations:** 1 Université de Poitiers, Faculté de Médecine et Pharmacie, 86000 Poitiers, France; 2 CHU de Poitiers, Service d’oncologie et Hématologie Pédiatrique, 86021 Poitiers, France; 3 CHU de Poitiers, Département des Agents Infectieux, 86021 Poitiers, France; 4 Université de Poitiers, INSERM U1070 Pharmacologie des Agents Antimicrobiens et Antibiorésistance, 86022 Poitiers, France

**Keywords:** *Corynebacterium gottingense*, bacteremia, sepsis, 16S rDNA sequencing, phylogenetic analysis

## Abstract

*Corynebacterium gottingense* is a Gram-positive bacillus that has not been reported as pathogenic in pediatric patients. Herein, a case of catheter-associated bloodstream infection by *C. gottingense* in a 13-year-old immunocompromised child with febrile neutropenia induced for osteosarcoma is reported. The species was identified by Sanger sequencing of the 16s rRNA sequence of the bacterial strain and was compared phylogenetically with published sequences. As suggested in the literature, the presented strain was multi-susceptible, particularly to amoxicillin. The patient was treated with piperacillin/tazobactam for seven days in the context of a urinary co-infection, resulting in resolution of fever within 48 h and then relaunched with oral amoxicillin for 3 days (for a total of 10 days of antibiotic therapy). Phylogenetic analyses based on 16S rDNA demonstrated the complexity of the genus *Corynebacterium* spp. but failed to demonstrate a direct benefit in predicting clinical outcome based on this single information.

## 1. Introduction

*Corynebacterium gottingense*, a Gram-positive, rod-shaped, catalase-positive, oxidase-negative bacterium, was first isolated from the blood of a patient with bacteremia of unknown origin at the Institute for Medical Microbiology in Gottingen, Germany, in 2012 [1].

The genus *Corynebacterium* was first described by Lechmann and Neumann in 1896 and later emended by Bernard et al. in 2020 [2]. This genus currently contains 136 validly published and correctly named species [3]. The genus *Corynebacterium*, considered saprophytic of skin and mucous membranes, is rarely considered pathogenic and is frequently found in the environment, including space flights [4]. Thus, *C. gottingense* is an extremely rare cause of human infections with only one case of bacteremia in the literature, reported by Atayasar et al. in 2017 [1]. This initial description of a human infection showed that this strain was susceptible to several drug classes but had reduced susceptibility or resistance to penicillin (including cephalosporins), erythromycin, and clindamycin [5].

In January 2018, Jani et al. described *Corynebacterium godavarianum* sp. Nov. (strain PRD07) isolated from the Godavari river in India in 2015 [6]. Wei et al. described, in May 2018, *Corynebacterium hadale* isolated from hadoplegic water of the New Britain Trench [7]. Heuristically, in 2020, Bernard et al. proposed that *C. godavarianum* and *C. hadale* were later heterotypic synonyms of *C. gottingense* based on 16S rRNA gene sequencing of strains with >99% similarity between them by 16s rRNA [2].

Here, the pediatric case of a 13-year-old boy who was admitted with febrile neutropenia in the setting of femoral osteosarcoma is reported. In addition to clinical and biological information, this case was confirmed by phylogenetic analyses. The latter was performed to explore the value of such information in determining the pathogenicity of such strains.

## 2. Case Report

A 13-year-old boy with a history of osteosarcoma of the left femur was admitted to the Department of Pediatrics and Oncology in Poitiers, France, for fever (>38.5 °C) in a setting of known multiple cytopenia (including leukopenia). Cytopenia was associated with the therapeutic management of his high-grade osteosarcoma with the SARCOME13/OS2016 post-operative protocol (cisplatine, ifosfamide, and adriamycine) covered by preventive treatment with cotrimoxazole.

On arrival at the hospital, his laboratory workup showed pancytopenia, i.e., anemia (hemoglobin 57 g/L), leukopenia (leukocytes 0.2 G/L), and thrombocytopenia (platelets 120 G/L) associated with a biological inflammatory syndrome (elevated inflammatory markers, including CRP 28 mg/L). Clinical examination revealed fever (with several peaks >39 °C). Given the clinical picture, preventive antibiotic therapy with ureidopenicillins (piperacillin combined with tazobactam, administered intravenously) was started immediately after taking blood cultures (from his port-a-catheter and peripheral veins) and urine samples.

**Figure 1:**
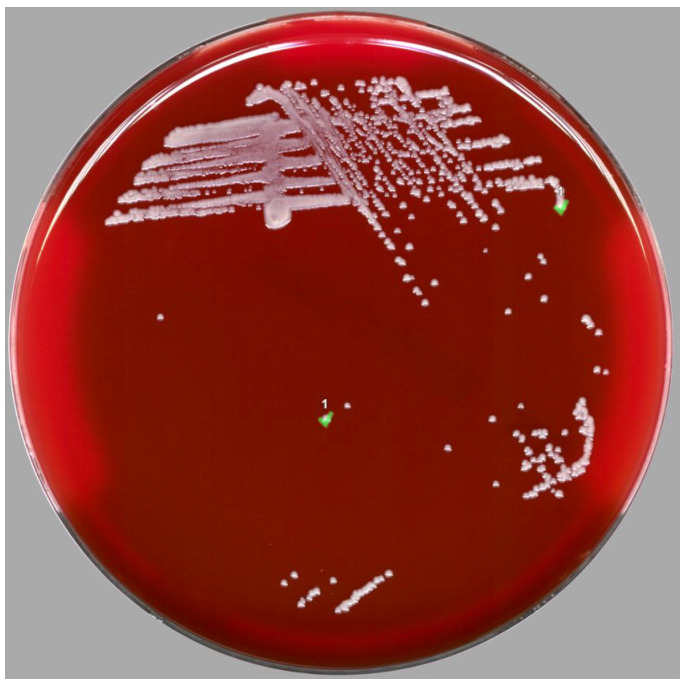
Colonies of *Corynebacterium gottingense* isolated on blood agar. Subcultured colonies (identified as “1”) could be considered as white (creamy) and were not hemolytic.

From a hematological standpoint, the patient received blood transfusions (one apheresis platelet concentrate and one red blood cell concentrate) and filgrastim (Zarzio^®^ 300 µg/dL). From an infectious standpoint, the urine examination (sampled due to urinary symptoms and positive screening by urine test strip) was positive for *Pseudomonas aeruginosa* 10^4^ CFU/mL, susceptible to piperacillin/tazobactam. Differential blood culture (i.e., anaerobic vial and aerobic vial blood cultures taken from the port-a-catheter (PAC) and two others from peripheral puncture) were detected as positive 46 h and 58 h after collection, respectively. Subculture (Figure 1) allowed the diagnosis of port-a-catheter infection by a *Corynebacterium* using MALDI-TOF (Vitek MS, bioMérieux, Marcy-l’étoile, France) but remained hesitant at the species level (suggesting different species with similar scores), justifying prospective rendering as *Corynebacterium* sp. before further investigations by molecular biology.

Even before species identification, the antibiotic susceptibility profile (AST) of this bacterium was determined using the disk diffusion method and the eTest (bioMérieux) according to the EUCAST/CA-SFM April 2021 recommendations. The AST profile revealed multi-susceptibility to all antibiotics tested, in particular, to amoxicillin (MIC = 0.38 µg/mL), ciprofloxacin, cotrimoxazole, rifampicin, linezolid, tetracycline, and vancomycin, but with two resistances (Penicillin G and clindamycin).

The patient was not afebrile 48 h after initiation of intravenous empiric antibiotic therapy (piperacillin/tazobactam), which was maintained for seven days to cover both a Pseudomonas aeruginosa urinary tract infection and a *Corynebacterium* sp. bacteremia. A three-day course of oral amoxicillin was introduced for a total of ten days of antibiotic therapy. It should be noted, in view of the very good improvement of the patient, that this PAC-onset bacteremia did not require removal or antibiotic locking of the PAC. Finally, as the patient had received his last course of chemotherapy, no further use of the PAC was considered.

Retrospective final identification of the organism involved as *Corynebacterium gottingense* was obtained by Sanger sequencing of the V1–V3 region of the 16S rRNA as described in another case report by our bacteriology department [8,9]. This sequence was analyzed using NCBI reference sequences (BLASTn) and BIBI-IV, and phylogenetic analysis was performed using Seaview V4 [10,11,12,13].

## 3. Results

**Figure 2:**
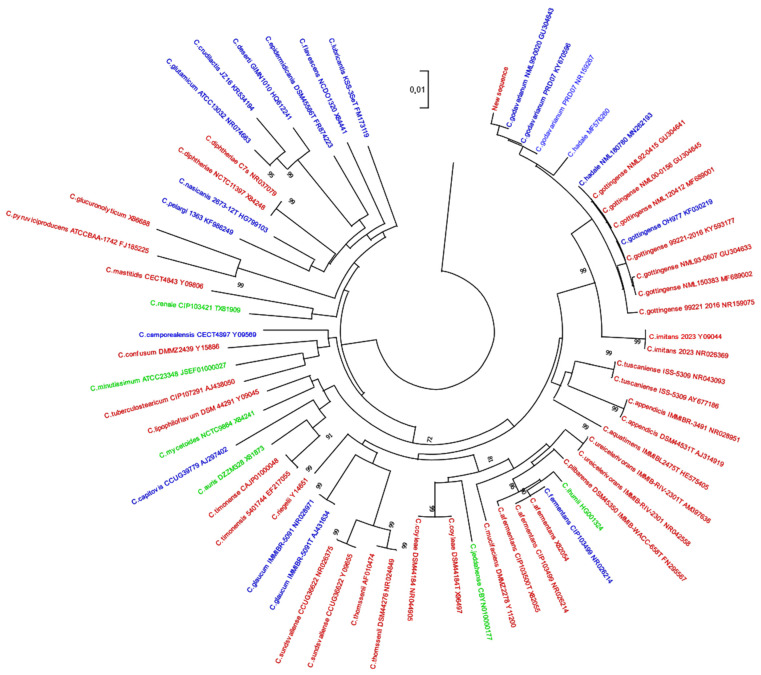
Phylogenetic analysis of *Corynebacterium* spp. identified. The sequences of isolated strains of human origin are shown in red and considered as pathogenic. The sequences of isolated strains of human origin are shown in green and considered as non-pathogenic. The sequences of isolated strains of environmental and/or non-human origin are shown in blue.

The resulting sequence of the bacterial strain (deposited on GenBank under accession number OP897049) was compared to sequences published in the literature at the time of writing (December 2022). Evolutionary history was inferred using the maximum likelihood method (Figure 2) [7]. Phylogenetic analysis revealed proximity between the new sequence and *Corynebacterium godavarianum, C. gottingense,* and *C. hadale*.

The evolutionary history was inferred by using not only the maximum likelihood method but also the Kimura 2-parameter model. The tree with the highest log likelihood (−24,084.70) is shown. The percentage of trees in which the associated taxa clustered together is shown next to the branches. Initial trees for the heuristic search were obtained automatically by applying neighbor-joining and BioNJ algorithms to a matrix of pairwise distances estimated using the maximum composite likelihood (MCL) approach and then by selecting the topology with a superior log likelihood value. The tree was drawn to scale, with branch lengths measured in the number of substitutions per site. This analysis involved 71 nucleotide sequences. All in all, there were 6205 positions in the final dataset. Evolutionary analyses were conducted in MEGAv11 [14].

The sequences were represented according to the origin of the strains. Three sequences isolated from strains of human origin and considered non-pathogenic are shown here. Other sequences from isolated strains of human origin considered pathogenic and from strains isolated from environmental origin are also represented. The trees of these partial 16S DNA sequences were not sufficient to classify these strains according to clinical outcome or bacterial strain origin (Figure 2).

## 4. Discussion

To the best of our knowledge, the present article is the first description of a case of *Corynebacterium gottingense* bacteremia described in a pediatric patient. Moreover, this is the first reported case in France.

The genus *Corynebacterium* could be part of the human commensal flora that may or may not be responsible for bacteremia in an opportunistic manner. While this genus includes the agent responsible for diphtheria (*Corynebacterium diphteriae* complex, responsible for a potentially fatal infection caused by this toxigenic strain) and while *C. jeikeium* and *C. urealyticum* are involved mainly in bacteremia and urinary tract infections, *C. gottingense* has been reported in only one case of bacteremia [15,16,17,18]. The context of the infection has not been described in the clinical data either. Although it was initially reported in bacteremia, it was subsequently isolated from the environment, once from a river and a second time from deep sea water. Therefore, this case could be considered as the first reported pediatric case of *C. gottingense* bacteremia in the literature.

In this case, the portal of entry for the infection was presumed to be the PAC, given the differential time of positivity (more than twelve hours) between PAC installation and the peripheral blood cultures before the peripheral puncture.

Moreover, this case demonstrated the rapid efficacy of antibiotic therapy in the absence of sepsis or shock. Indeed, the patient rapidly became apyretic after only 48 h of empiric antibiotic therapy with piperacillin/tazobactam. Moreover, evolution was favorable with no other positive blood cultures in the patient’s continuing clinical history. In this context, the PAC was not removed and did not require any particular action to treat this entry point (antibiotic lock, surgical intervention to remove the PAC …).

The *Corynebacterium* described in this pediatric case follows the modified description of *Corynebacterium gottingense* depicted by Bernard et al. in 2020 (Table 1). Indeed, the strain was isolated from aerobic and anaerobic blood cultures, and was susceptible to several classes of drugs including amoxicillin, ciprofloxacin, cotrimoxazole, rifampicin, linezolid, tetracycline, and vancomycin, but had natural resistance to penicillin G and clindamycin.

Taxonomic analyses of the V1–V3 regions of the 16S rDNA of the isolated strain demonstrated the complexity of the genus *Corynebacterium* (Figure 2). Nevertheless, the data did not allow classification of the strain as pathogenic or non-pathogenic according to this phylogenetic classification alone [19]. This information suggests that more information is needed to improve the knowledge of the genus in order to differentiate those involved in pathogenesis from those that are only considered as normal constituents of the human skin microbiome [4].

## 5. Conclusions

This case of *Corynebacterium gottingense* bacteremia in an immunocompromised pediatric patient underscores the importance of sequencing new strains of *Corynebacterium* sp. to reference pathogenic strains in a genus such as *Corynebacterium*, which is predominantly described in the environment. This case suggests the benefit of following the emergence of new pathogens in the genus *Corynebacterium*.

## Figures and Tables

**Table 1:** Comparison between the different strains of *Corynebacterium* spp. to an emended description of *Corynebacterium gottingense* in 2020.

Classification	*C. godavarianum*, 2018 [6]	*C. hadale*, 2018 [7]	*C. gottingense*, 2017 [1]	Emended Description, 2020 [2]	Present Description
Phylum	*Actinobacteria*				
Family	*Corynebacteriaceae*				
Genus	*Corynebacterium*				
Reference Strain	NR_159267	MF576260	NR_159075	-	OP897049.1
Isolation	Godavari river in India	Deep sea water in the New Britain trench	Blood of clinical patient with bacteraemia in a German city	-	Blood of clinical patient with bacteraemia in a French city
Morphology					
- Cell shaped	Rod-shaped				
- Motility	No	-	-	-	-
- Gram stain	Positive	-	-	-	Positive
Oxygen tolerance	Aerobic	Anaerobic	Aero-anaerobic	-	Aero-anaerobic
Catalase	Positive	-	-	-	Positive
Length	1 µm	1.3 µm	-	-	-
Diameter	0.5 µm	0.57 µm	-	-	-
Colony color on blood agar	Creamy-whitish and non-translucent	Ivory-white	White cream	-	White cream
Hemolysis on agar supplemented with 5% sheep blood	Non haemolytic	-	-	-	Non haemolytic
Culture and growth conditions					
Temperature	37° (range 25–40 °C)	Optimal 35 °C (range 25–41 °C)	37°	35–37°	35–37°
pH	Optimal: 7.0 (range 6–11)	-	-	6–7	-
NaCl	Optimum 1% (tolerance up to 11%)	Optimal 4% 0–10%	-	0–4%	-
Enzyme production					
Catalase		+	+	+	+
Oxidase	-	-	-	-	-
Gelatinase	-	/	/	-	/
Nitrate reductase	-	-	-	-	-
H2S	-	/	/	/	/
Acid production as the only carbon substrate from					
Glucose	-	/	+	+	/
Ribose	+	/	+	+	/
Maltose	+	/	+	+	/
Antibiotic Susceptibility Testing (AST)					
Susceptible	Amikacin, cefixime, cefotaxime, ceftriaxone, chloramphenicol, ciprofloxacin, colistin, co-trimoxazole, furazolidone, gentamicin, levofloxacin, netillin, norfloxacin, rifampicin, streptomycin, tetracycline and vancomycin	-	-	-	Amoxicillin, ciprofloxacin, cotrimoxazole, rifampicin, linezolid, tetracycline and vancomycin
Resistance	Amoxicillin, co-amoxiclav, cefpodoxime, and clindamycin	-	-	-	Penicillin G and clindamycin

## Data Availability

The resulting sequence of the bacterial strain was deposited on GenBank under accession number OP897049.
